# Available ablation energies to treat cT1 renal cell cancer: emerging technologies

**DOI:** 10.1007/s00345-018-2546-6

**Published:** 2018-11-17

**Authors:** P. J. Zondervan, M. Buijs, D. M. De Bruin, O. M. van Delden, K. P. Van Lienden

**Affiliations:** 1 Department of Urology, Amsterdam UMC, University of Amsterdam, Meibergdreef 9, 1105 AZ Amsterdam, The Netherlands; 2 Department of Biomedical Engineering and Physics, Amsterdam UMC, University of Amsterdam, Meibergdreef 9, 1105 AZ Amsterdam, The Netherlands; 3 Department of Radiology, Amsterdam UMC, University of Amsterdam, Meibergdreef 9, 1105 AZ Amsterdam, The Netherlands

**Keywords:** Cryoablation (CA), Radiofrequency ablation (RFA), Small renal masses (SRM), Renal cell cancer (RCC), Microwave ablation (MWA), Irreversible electroporation (IRE), Stereotactic ablative radiotherapy (SABR)

## Abstract

**Purpose:**

An increasing interest in percutaneous ablation of renal tumors has been caused by the increasing incidence of SRMs, the trend toward minimally invasive nephron-sparing treatments and the rapid development of local ablative technologies. In the era of shared decision making, patient preference for non-invasive treatments also leads to an increasing demand for image-guided ablation. Although some guidelines still reserve ablation for poor surgical candidates, indications may soon expand as evidence for the use of the two most validated local ablative techniques, cryoablation (CA) and radiofrequency ablation (RFA), is accumulating. Due to the collaboration between experts in the field in biomedical engineering, urologists, interventional radiologists and radiation oncologists, the improvements in ablation technologies have been evolving rapidly in the last decades, resulting in some new emerging types of ablations.

**Methods:**

A literature search was conducted to identify original research articles investigating the clinical outcomes of new emerging technologies, percutaneous MWA, percutaneous IRE and SABR, in patients with primary cT1 localized renal cell cancer.

**Results:**

Due to the collaboration between experts in the field in biomedical engineering, urologists, interventional radiologists and radiation oncologists, the improvements in ablation technologies have been evolving rapidly in the last decades. New emerging technologies such as microwave ablation (MWA), irreversible electroporation (IRE) and stereotactic ablative radiotherapy (SABR) seem to be getting ready for prime time.

**Conclusion:**

This topical paper describes the new emerging technologies for cT1 localized renal cell cancer and investigates how they compare to CA and RFA.

## Introduction

With the increasing use of cross-sectional imaging, the subsequent rise in incidence of SRMs, the evolution of new local ablative technologies and the aging population, percutaneous ablation of localized RCC in both fit and unfit patients is gaining more interest. As in the early days the different guidelines advised to offer focal therapy only for the elderly or unfit patient, and until today the EAU guideline still does, the AUA guideline on the contrary is more progressive: they advise considering thermal ablation as an alternate approach for the management of cT1a renal masses < 3 cm in size [1, 2].

Focal therapy can be very attractive for patients for several reasons. Image-guided ablation is minimally invasive, allows for a quick patient recovery, short hospital stay, fewer complications and smaller reduction in renal function as compared to surgery [3]. CA and RFA are the most studied techniques to date with the longest outcomes reported and both are therefore advised as the designated modalities for SRMs in the various guidelines [1–4]. Yet, CA and RFA have their drawbacks and limitations. New competing emerging technologies such as MWA, IRE and stereotactic ablative radiotherapy (SABR) are increasingly used or under clinical investigation and appear promising.

The scope of this topical paper is to describe these emerging technologies and assess their potential roles as compared to the current standard techniques CA and RFA. A literature search was conducted to identify original research articles investigating the clinical outcomes of percutaneous MWA, percutaneous IRE and SABR in patients with primary cT1 localized renal cell cancer.

## Microwave ablation (MWA)

MWA is a thermal ablation technique, which uses electromagnetic waves through one or multiple antennas. The electromagnetic microwaves agitate water molecules in the surrounding tissue, producing friction and heat, causing cell death by coagulative necrosis [5].

One randomized controlled trial was published in 2012 comparing open partial nephrectomy (OPN), laparoscopic partial nephrectomy (LPN), open MWA and laparoscopic MWA. Besides the fact that there was less blood loss, fewer complications and less decline in postoperative renal function in favor of MWA, no difference was found in local recurrence-free survival with a median follow-up of 32 months. The major limitation of this study was the small number of inclusions: 48 underwent microwave ablation [6]. Regarding studies on percutaneous MWA, only case series have been published so far. Although concerns were raised initially about higher local recurrence and complication rates for MWA in comparison to RFA and cryoablation, more recent data have shown that outcomes are in fact comparable.

We selected original research studies with > 50 cases of percutaneous MWA in patients with cT1 localized RCC, and the results of seven studies are listed in Table 1 [7–13].

**Table 1:** Summary of studies assessing percutaneous microwave ablation in localized cT1 primary renal cell carcinoma with > 50 cases

Study	Design	Level of evidence^a^	Technique	Number of cases	Charlson Comorbidity Index	Tumor size mean (cm)	Complications	Follow-up (months) median	Renal function decrease (%)	Residual disease (%)	Local recurrence (%)	Metastasis (%)	DFS (%)	OS (%)	CSS (%)
Li [7]	Prospective	3	US guided, general anesthesia	83	NR	3.2 ( ± 1.6)	No severe complications	26(3–74) CEUS and CT/MRI	NR	18.3% CEUS and CT/MRI and pathology proven	8.4% CT/MRI, CEUS and pathology proven	NR	NR	96.4%	NR
Moreland [8]	Retrospective	3	US and CT guided, general anesthesia	53	3 (median)	2.6 (0.8–4.0)	11.3%	8(6–9) CT/MRI	1,1%	No residual CT/MRI	No local recurrence CT/MRI	No metastasis	NR	NR	NR
Yu [9]	Retrospective	3	US guided, moderate sedation	105	32% CCI > 2	2.7 (0.6–4.0)	3.2%	25.8(3.7–75.2) CEUS/CT/MRI	3.2%	13.3% CEUS/CT/MRI	0.95% CT	2.9%	97% (5 years)	82.6% (5 years)	97% (5 years)
Dong [10]	Retrospective	3	US guided, general anesthesia	105	NR	2.9 (0.6–6)	24.8%	25 (1.13–93.23) CEUS and CT/MRI	8.4%	NR	NR	NR	NR	NR	NR
Ierardi [11]	Retrospective	3	CEUS, CT guided or US guided	58	NR	2.36 ± 0.93	8.6%	25.7 (3–72)CT	NR	7% CT	15.7%	NR	87.9% (5 years)	80.6% (5 years)	96.5%
Chan [12]	Retrospective	3	CT guided, general anesthesia	84	NR	2.5	4.8%	24 CT	7%	8% CT	3.8%	2.4%	95% (2 years)	97% (2 years)	NR
Klapperich [13]	Retrospective	3	CT-guided, general anesthesia	96	3.6 ± 1.5 (mean)	2.6 (1.2–4)	11%	15 (6–20) CT/MRI	3.3%	No residual CT/MRI	1% Pathology proven	No metastasis	NR	91% (3 years)	100% (3 years)

Residual tumor is defined as unablated residual tumor at initial follow-up imaging

Recurrence is defined as the appearance of tumor at the edge or in the ablation zone

*DFS* disease-free survival, *OS* overall survival, *CSS* cancer-specific survival, *NR* not reported. *CCI* Charlson Comorbidity Index

^a^Level of evidence Oxford 2011 [14]

All of the studies were level of evidence 3, mainly retrospective observational studies.

MWA was performed with either ultrasound or CT guidance. The duration of ablation across the studies was mainly short (5–22.5 min), while the total procedure times took longer (22.5–45 min). The number of probes used ranged from 1 to 2, with one antenna used when tumor size < 2 cm, two antennas used when tumor size ≥ 2 cm, and three antennas were used when tumor size was > 3 cm. With mean tumor sizes ranging from 2.3 to 3.2 cm, a low percentage of complications was reported (3.2–24.8%). Complications reported mainly consisted of hematuria, perirenal hematomas, or urinoma. The functional results after MWA showed only a decrease in renal function ranging from 1.1 to 8.4% across the studies.

Concerning oncological outcomes, residual disease was reported ranging from 0 to 18.3%, and local recurrences ranged from 0 to 15.7%. With median follow-up periods ranging from 8 to 26 months, a low percentage of metastases was reported (0–2.9%). Overall survival ranged from 80.6 to 97%. DFS ranged from 87.9 to 97%, and CSS ranged from 97 to 100%. The major drawback is that any residual or recurrence during follow-up was mostly not proven with pathology but only on imaging. The limitation of these studies is their retrospective nature, the relatively short follow-up, and the small tumor sizes. Although data on MWA seems promising, they have currently not reached the long-term outcomes of the thoroughly studied modalities using RFA and CA [1–3].

Potential advantages of MWA are shorter ablation and procedure times as compared with RFA and CA [15], less influence by the heat-sink effect of the blood circulation as compared to RFA [5, 16] and the potential of MWA to achieve larger ablation zones than RFA (Table 4). In future, MWA can potentially compete with CA for larger (cT1b) lesions. A potential disadvantage of MWA is the unpredictability of the ablation zone as compared to CA, but this may be resolved as technology improves.

## Irreversible electroporation (IRE)

IRE is a novel focal ablation method based on the principle of electroporation of the cell membrane. By using ultrashort high-voltage electrical pulses, it causes nanopores in the cell membrane and consequently an increased cellular permeability causing cell death by apoptosis [17]. Although IRE is supposed to be a non-thermal ablation modality, a secondary rise in temperature has been shown [18]. Whether thermal damage accompanying the non-thermal damage is of any relevance is still a matter of debate. The most characteristic feature of IRE is that the lesions show a sharp demarcation zone between ablated and non-ablated tissue, making IRE particularly useful for planning more precise tumor ablation while preserving surrounding tissue. This can be an advantage in tumors in difficult locations or with a complex shape.

Experience with IRE for renal tumors is limited. We selected original research studies for the safety and feasibility of IRE in primary RCC; the results of seven studies are listed in Table 2 [19–26].

**Table 2:** Summary of studies assessing irreversible electroporation (IRE) in localized cT1 primary renal cell carcinoma

Study	Design	Level of evidence^a^	Technique	Number of cases	Charlson Comorbidity Index	Tumor size mean	Complications	Follow-up (months)	Renal function decrease	Residual disease (%)	Local recurrence (%)	Metastasis (%)	DFS (%)	OS (%)	CSS (%)
Pech [19]	Phase I, prospective	3	Open IRE, general ANS	6	NR	2.7 (range 2.0–3.9)	No complications	NR	NR	NR	NR	NR	NR	NR	NR
Thompson [20]	Prospective cohort	3	Percutaneous IRE US or CT guided, general ANS	10 (7 patients)	NR	2,2 (range 1.6–3.1)	Cardiac arrhythmias, partial ureteric obstruction	NR CT	NR	2/7 residual at 3 months CT	NR	NR	NR	NR	NR
Trimmer [21]	Retrospective	4	Percutaneous CT-guided IRE, general ANS	20	NR	2.2 ± 0.7	Minor: perinephric hematomas, pain, urinary retention	1 year in 30% available CT/MRI	Creatinine −0.04 mg/dL after 6 weeks	2/20 residual at 6 months CT/MRI	1/20 recurrence at 1 year CT/MRI	NR	NR	NR	NR
Diehl [22]	Retrospective	4	Percutaneous CT-guided IRE, general ANS	7 (5 patients)	NR	2.44 (1.5–3.8)	2 minor complications: hematuria, AKI	Mean 6.4 (3–11)	Creatinine −2.75 mL/min in 3 months	No residual MRI	NR	NR	NR	NR	NR
Wendler [23–25]	Phase 2a, prospective	3	Percutaneous CT-guided IRE, followed by resection, general ANS	8 (7 patients)	Ranging 0–2	Mean 2.2 (1.5–3.9)	Hematuria7/7, perirenal hematoma 2/7, pain 7/7	Mean 25(15–36)	NR	3/8 residual Pathology proven.	1/8 recurrence	NR	NR	NR	NR
Canvasser [26]	Retrospective	3	Percutaneous CT-guided IRE, general ANS	42 (41 patients)	NR	Mean 2.0 (1.0–3.6)	22% Clavien grade I: perinephric hematoma, urinary retention,	Mean 22 (SD 12.4)	eGFR −6 mL/min	3 failures/42 CT	2/42 recurrence CT	NR	NR	NR	NR
Buijs [27] and submitted	Prospective, phase 2b/3	3	Percutaneous CT-guided IRE, general ANS	10	Mean CCI corrected for age: 7	2.2 (1.1–3.9)	1 × grade 3: obstruction ureter due to blood clot 1, 1 × grade 2: pyelonephritis 3 × grade 1: 1 perinephric hematoma, 1 painful voiding, 1 hematuria	Mean 6 (3–12)	2,6% decrease after 1 year	1/10 residual CT	No recurrences	NR	NR	NR	NR

Residual is defined as enhancement reported at the first imaging after IRE in the ablation zone

Recurrence is defined as new enhancement after a period of non-enhancement in or at the edge of the ablation zone

*AKI* acute kidney insufficiency, *US* ultrasound, *CT* computo-tomography, *ANS* anesthesia, *DFS* disease-free survival, *OS* overall survival, *CSS* cancer-specific survival, *NR* not reported, *CCI* Charlson Comorbidity Index

^a^Level of evidence, Oxford 2011 [14]

All of the studies selected were level of evidence three or four (Table 2). Pech et al. demonstrated the feasibility and safety of IRE in ‘an ablate and resect’ clinical Phase 1 study in six patients [19], while Thompson performed IRE in ten patients [20]. Two retrospective studies performed by Trimmer [21] and Diehl [22] investigated the feasibility and short-term functional and oncologic outcomes after percutaneous IRE of 20 and 7 renal tumors, respectively. Wendler et al. have done extensive work on IRE in the IRENE study, in which patients underwent percutaneous CT-guided IRE and 4 weeks later radical or partial nephrectomy [23–25]. Canvasser et al. published about 42 renal tumors for which CT-guided IRE was performed [26]. Buijs et al. submitted a paper presenting the preliminary results of ten patients who underwent percutaneous IRE [27].

Percutaneous IRE was mainly performed CT-guided. The numbers of needles used across the studies ranged from 3 to 4. If reported, procedure times ranged from 53 to 203 min, dependent on the tumor size and complexity of needle positioning and the shape needed to get the correct ablation zone. Mean tumor sizes ranged from 2.0 to 2.7 cm.

Mainly minor complications were reported and consisted of perinephric hematomas, post-procedural pain and urinary retention. The major complications reported in two studies were transient gross hematuria in a hilar tumor and stage 1 acute kidney failure [22, 27]. Renal function decline was minimal, but was not always reported. Wendler et al. showed preservation of the urine collecting system: the urothelium showed signs of regeneration after 28 days, while the tumor and parenchyma showed clear necrosis and permanent cell destruction [25].

Follow-up was available in five of seven studies: the mean follow-up ranged from 6 to 25 months. A high percentage of residual disease was reported (range 7–37.5%). Some of the authors suggested that residual tumor was most likely the result of probe malpositioning [21, 26, 27]. Wendler et al. have done extensive work on IRE in the IRENE study in which patients underwent percutaneous CT-guided IRE and 4 weeks later radical or partial nephrectomy. They demonstrated microscopic residual tumor cells in three out of eight biopsy-proven RCC cases. Although they questioned the clinical relevance of the microscopic tumor residues remaining in the non-viable ablation region, this still offers a possibility of repeat ablation in their opinion [23, 24]. In their study, one patient showed local recurrence after 1 year. The studies that reported recurrences showed percentages ranging from 0 to 12.5% (Table 2).

Little is published concerning post-procedural imaging in these studies. Some showed slightly larger hypodense area of the ablation zone, compared to the original tumor, and surrounding areas of enhancement in the perinephric fat [21]. Diehl et al. showed a progressive, significant decrease in treated tumor signal intensity on follow-up imaging, suggesting a treatment response rate of 100% at a mean follow-up of 6.4 months [22].

The limitation of some of the studies was the low number of biopsy-proven tumors treated.

A major drawback of IRE is the need for deep muscle relaxation and ECG synchronized pulsing during general anesthesia. The pulsatile application of electricity with a high current of around 20–50 A and a voltage of 500–3000 V is a major challenge for the anesthesiologist; it can give possible triggering of cardiac arrhythmias and severe muscle contractions or epileptic seizures [28].

Although IRE seems feasible and safe, these results are preliminary and need technical improvement to ensure oncological results. Furthermore, the remaining questions are how do we know at what time the effect of IRE is successful, and how to interpret the imaging during follow-up, and what is the best imaging modality to use? All these questions are still remaining, and need further investigation. IRE is promising in selected cases, but the main disadvantages are the need for general anesthesia with deep muscle relaxation and long procedure times caused by complex positioning of needles.

## Stereotactic ablative radiotherapy (SABR)

Radiotherapy has been settled as a palliative treatment option in the armamentarium of the urologist in the metastasized setting for renal cell cancer. In the past, conventional radiotherapy had a limited role in the treatment of primary RCC largely due to the supposed radioresistance of RCC. In retrospect, this is mainly caused by the fact that too low doses were given. Due to the availability of new technologies that deliver high-dose stereotactic ablative radiotherapy, there has been a shift toward possible treatment options for primary RCC with curative intent. To date, SABR is mainly chosen as a treatment option for patients who are at high risk for anesthesia. In addition, high-dose radiation seems to have an immunogenic effect in patients with RCC; the intense localized radiation provided by SABR would drive the release of antigens by tumors, inducing a tumor-specific T cell response [29].

We selected original research studies for primary RCC who underwent SABR with > 20 cases; besides a systematic review, only two studies were selected (Table 3).

**Table 3:** Summary of studies assessing stereotactic ablative radiotherapy (SABR) in localized primary renal cell carcinoma

Study	Design	Level of evidence^a^	Technique	Number of cases	Charlson Comorbidity Index	Tumor size(cm)	Complications	Follow-up (months)	Renal function decrease (%)	Residual disease (%)	Local recurrence (%)	Metastasis (%)	DFS (%)	OS (%)	CSS (%)
Siva [30]	Systematic review	2	SABR 3, 4, and 5 fraction approaches used. Mostly used: 40 Gy over 5 fractions.	126	NR	NR, but no size restrictions	Weighted rate of severe toxicity 3.8% Weighted rate of minor toxicity 21.4% Reported fatigue, nausea, radiation dermatitis and enteritis	Median/mean ranged from 9 to 57.7	NR	NR	Weighted local control 93.9% (range 84–100%) CT/MRI	NR	NR	NR	NR
Siva [31]	Prospective	3	Single 26 Gy (for tumor size ≤ 5 cm) versus 3 fractions of 14 Gy (for tumor size > 5 cm) SABR	37	76% of the patients had CCI ≥ 6	Median 4.8 (2.1–7.5)	78% Grade1–2 toxicity 3% Grade 3 toxicity Reported: fatigue chest wall pain, nausea	Median 24	11 mL/min eGFR in 1 year	NR	2 years freedom for local progression 100% CT/MRI	NR	2 years freedom from distant progression 89% (CI 78–100)	92% (2 years)	NR
Siva [32]	Multi-institutional pooled analysis	3	Both single-, and multi-fraction SABR included. Mean Gy 25 (14–70)	223	NR	Mean 4.36 (± 2,77)	35.6% Grade 1–2 toxicity 1.3% Grade 3–4 toxicity Reported: nausea, bowel toxicity	Median 30	Mean decrease in eGFR 5.5 ± 13.3 mL/min	NR	1.4% Local control: 97.8% at 2 years and 4 years CT/MRI	7.2%	PFS 77.4% (2 years) 65.4% (4 years)	82.1% (2 years) 70.7% (4 years)	95.7% (2 years) 91.9% (4 years)

Local recurrence was defined using Response Evaluation Criteria in Solid Tumors, version 1.0

Residual was not defined and not reported across all studies

*ANS* anesthesia, *DFS* disease-free survival, *OS* overall survival, *CSS* cancer-specific survival, *NR* not reported. *CCI* Charlson Comorbidity Index

^a^Level of evidence, Oxford 2011 [14]

Siva et al. performed a systematic review on SABR for RCC with no tumor size restrictions in 2012. In total, ten publications describing 126 patients reported treatment with one to six fractions of SABR [30]. Recently the same group published a prospective study in which 37 patients with inoperable primary RCC underwent SABR. Tumors of < 5 cm received one single SABR of 26 Gy delivered, while in tumors ≥ 5 cm 42 Gy was delivered in three fractions [31]. Furthermore, safety, efficacy and survival were assessed in a multi-institutional setting in 223 patients from nine institutions. Both single-fraction SABR and multi-fraction SABR were given [32].

Tumor sizes ranged from 2.1 to 7.5 cm in the studies, representing not only cT1 but also cT2a tumors [31, 32]. Treatment-related toxicities were defined using Common Terminology Criteria of Adverse Events: severe toxicity ranging from 1.3 to 3.8% grade 3–4. Grade 1 toxicity ranged from 21.4 to 78%. Mainly fatigue, nausea, radiation dermatitis and enteritis were reported. There was a relatively minor decrease in eGFR after SABR (5.5–11 mL/min) even when considering that tumor size was larger than that in the studies performing IRE of MWA. With a median follow-up ranging from median 9–57 months across the studies, the local control was excellent for patients with comorbid conditions, especially because the reported CCI was > 6 in 76% of the patients [31].

Interestingly, single-fraction SABR was associated with better progression-free survival and cancer-specific survival. An interesting observation in the multi-institutional study was that patients receiving single-fraction SABR appear to be less likely to progress distantly or to die of cancer, something not fully understood [32]. A potential explanation could be the enhanced abscopal effect of distant tumor cell eradication because of single-fraction irradiation. An alternative hypothesis is that during fractionated radiotherapy, circulating tumor cells are released in the circulation, which may still be viable after smaller doses of fractionated radiotherapy [32]. Concluding from these series, cT1 primary tumors, and even cT2 tumors, can be ablated in the unfit patient who is unable to undergo anesthesia with promising results.

## Considerations

In daily practice, it is challenging to decide which treatment modality is best for cT1 localized RCC. With the increasing incidence of SRMs and the aging population not fit for surgery, an increased demand for ablations may be expected. In addition to this, we are confronted with the fit patient requesting for ablation, while for these patients partial nephrectomy is still advised. Following the paradigm of shared-decision making, it could be that even fitter patients will choose for focal therapy in the future. The AUA guideline advises considering thermal ablation as an alternate approach for the management of cT1a renal masses < 3 cm in sizes (2), while the EAU still advises offering CA or RFA only to elderly and/or comorbid patients with SRMs (1). Both CA and RFA are the designated types of ablation advised by the different guidelines [2, 3]. Whichever ablative technique is chosen, counseling should always include information regarding the likelihood of tumor persistence or local recurrence. Furthermore, a percutaneous approach is preferred over a surgical approach whenever feasible to minimize morbidity [2]. Recent studies on the use of ablation in SRMs confirmed a decrease in the use of laparoscopic ablations [33]. Comparing RFA and CA, conflicting data regarding efficacy and oncological outcomes have been described [34–36]. In practice, RFA seems to be faster and cheaper, while CA takes more time, is more expensive, but can potentially be more precise in monitoring the ‘ice-ball’ during the procedure. Furthermore, CA seems to be more effective in cT1b tumors [37]. The majority of complications associated with ablation are minor. While CA has a higher chance for bleeding (up to 8%), RFA gives less bleeding complications as a result of its coagulative effect (up to 4%) [38]. For RFA, the major complication is urothelial damage leading to urinary leak or possible stricture (up to 4.8%) [39]. On the contrary, CA produced less harm to the collecting system or ureter (1–2%) [40, 41].

The advantage of CA is creating a large ‘ice-ball’, with multiple cryoneedles, in different sizes, which can be visualized during the procedure. This means real-time monitoring of the ablation is possible. RFA uses one or more probes, but the presumed ‘fire-ball’ cannot be visualized on CT during the procedure.

With the evolution of new technologies, MWA, IRE and SABR have made their entrance. While the body of evidence for MWA in renal masses is still limited, it could have some potential advantages in ablating larger lesions possibly even in a shorter time compared to RFA or CA [15]. As oncological data will mature in the coming years for MWA, they probably will become comparable to RFA and CA. While MWA is routinely used for SRM to date, IRE is still under investigation. IRE has proven to be feasible and safe in small series, but is by far not settled, and further research has to be done in the field. Even when IRE is to be introduced in clinical practice, it will solely be in selected cases: in centrally located tumors or when vital structures are very close and in patients who can undergo general anesthesia with muscle relaxation. SABR is safe with low toxicity, has no definite size limitation, is not limited by tumor location and is thought to promote antitumor immunity. Despite the fact that the optimal dose and fractionation regimens are not yet definite and oncological results are limited, it could be the sole solution in some selected cases [31, 32]. The immunogenic effect seen in SABR is also suggested in other ablation types and the combination with immunotherapy such as immune checkpoint inhibition could be promising in the future [42]. Further research in dose fractionation and oncological longer-term follow-up in SABR has to settle the treatment type for SRMs.

**Fig. 1:**
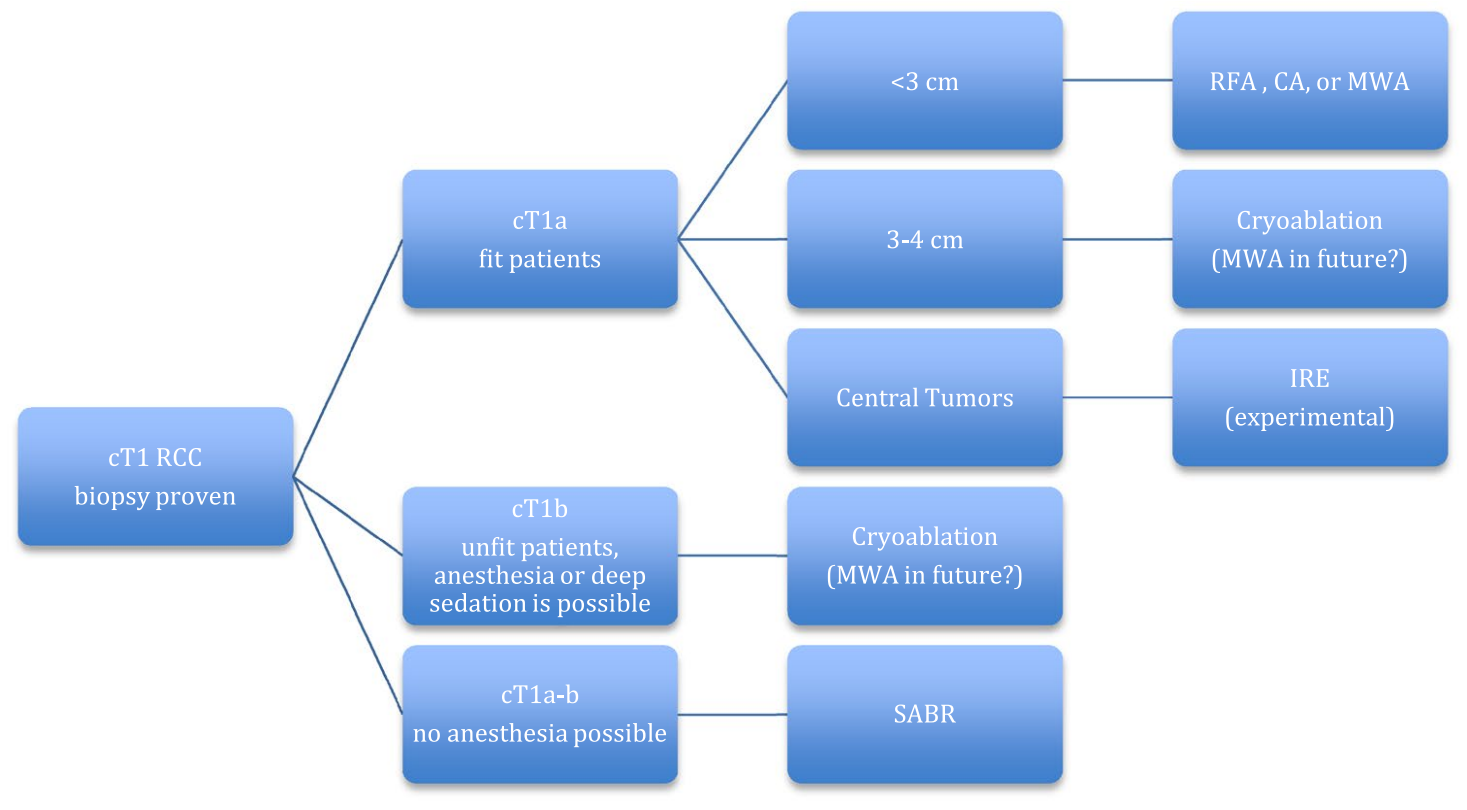
Flowchart for guiding decision making for type of ablation in cT1 localized RCC. For SRMs in fit patient, different types of ablation are possible (CA, RFA, MWA, IRE in selected cases), while for cT1a-b tumors in unfit patients CA or SABR is advised. SRMs < 3 cm are expected to be in the favorable prognostic group [43], for this reason a cut-off value of tumor size 3 can be used, as bigger lesions and cT1b lesions are best treated with CA [37]

The dilemma concerning which type of ablation should be used in selected cases is still unresolved. The type of ablation chosen for a cT1 renal cell cancer mostly depends on the experience and expertise of the urologist and interventional radiologist, and the available resources. The individual characteristics of each kidney tumor are different and make it necessary to adapt the type of ablation to this particular tumor. Proper case selection is very important when considering the use of ablative therapies. Furthermore, the condition of the patient is vital in deciding the possibilities for the type of ablation. The percutaneous approach is preferred over the laparoscopic because of fewer complications and its minimal invasiveness [44]. The different types of ablation available offer a solution to give direction to this decision-making process when considering their pros and cons when evidence is more sound (Table 4, Fig. 1).

**Table 4:** Practical pros and cons of different types of percutaneous ablation in cT1 RCC

	Pro	Con
RFA	Single needle possible. Coagulative properties. Can be done under deep sedation, general anesthesia not mandatory. Quicker than CA. Good evidence available	Heat-sink effect. No real-time monitoring of ablation zone. Limited size of ablation zone. Risk for urothelial damage
Cryoablation	Real-time monitoring of ablation zone possible. Large ablation size possible. Can be done under deep sedation, general anesthesia not mandatory. Good evidence available	Heat-sink effect. Multiple needles often required. Risk for bleeding. More time-consuming than RFA and MWA
MWA	Quicker than RFA and CA. Higher temperatures than RFA. Coagulative properties. Can be done under deep sedation, general anesthesia not mandatory	No real-time monitoring of ablation zone. Risk for urothelial damage. Limited evidence available
IRE	Direct post-procedural monitoring possible. No injury to surrounding structures. Well suited for centrally located tumors	General anesthesia with muscle relaxation and EKG triggering required. Multiple parallel placed needles required. More time-consuming than CA, RFA and MWA. No sound evidence available
SABR	Truly non-invasive. No anesthesia required. No size limit	Renal function impairment. No sound evidence available

## Conclusion

Image-guided percutaneous ablation for cT1 localized renal cell cancer has become a readily available and competing alternative for partial nephrectomy. Due to an expected increase in SRMs, an aging population, awareness of shared-decision making, patient preference and the rapid technical improvements in ablative therapies, this treatment modality will become much more relevant in the near future. Image-guided ablation is a good alternative for SRMs in fit patients, but seems also a good solution in cT1b tumors in the unfit patient. As new emerging technologies are rising to compete with their historical counter partners, we will need to carefully evaluate them. With the introduction of MWA, IRE and SABR, the near future will learn if these new emerging technologies are ready for prime time and how they will eventually settle in the palette of image-guided ablation for cT1 localized renal cell carcinoma.

## Abbreviations

RCC: Renal cell cancer
CA: Cryoablation
RFA: Radiofrequency ablation
SRMs: Small renal masses
MWA: Microwave ablation
IRE: Irreversible electroporation
SABR: Stereotactic ablative radiotherapy
eGFR: Estimated glomerular filtration rate
LRFS: Local recurrence-free survival
CSS: Cancer-specific survival
CT: Computed tomography
